# 2-Imi­niumyl-1,3-diazepane-4-carboxyl­ate

**DOI:** 10.1107/S1600536810049676

**Published:** 2010-12-04

**Authors:** Feng Yang

**Affiliations:** a School of Chemistry and Chemical Engneering, Guangxi Normal University, Guilin 541004, People’s Republic of China

## Abstract

The title compound, C_6_H_11_N_3_O_2_, is a cyclized derivative of l-arginine and the mol­ecule is a zwitterion with the positive and negative charge residing in the guanidinium and carboxyl­ate groups, respectively. The conformation of 1,3-diazepane ring is close to a twisted chair. One intra­molecular and three inter­molecular N—H⋯O hydrogen bonds stabilize the mol­ecular conformation and the crystal structure, respectively.

## Related literature

For related structures, see: Karapetyan (2008*a* ▶,*b* ▶).
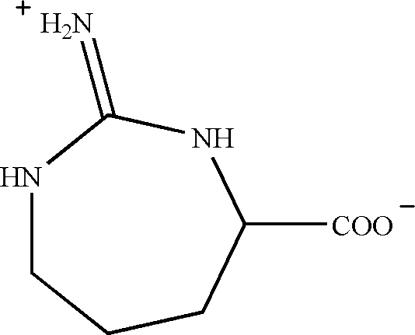


## Experimental

### 

#### Crystal data

C_6_H_11_N_3_O_2_
*M* *_r_* = 157.18
Orthorhombic, 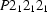

*a* = 6.1740 (3) Å
*b* = 8.7979 (5) Å
*c* = 14.2036 (7) Å
*V* = 771.51 (7) Å^3^
*Z* = 4
Mo *K*α radiation
μ = 0.10 mm^−1^
*T* = 293 K
0.23 × 0.15 × 0.10 mm

#### Data collection

Siemens SMART CCD area-detector diffractometer
Absorption correction: multi-scan (*SADABS*; Sheldrick, 1996 ▶) *T*_min_ = 0.773, *T*_max_ = 1.000
3426 measured reflections
834 independent reflections
694 reflections with *I* > 2σ(*I*)
*R*_int_ = 0.026

#### Refinement

*R*[*F* ^2^ > 2σ(*F* ^2^)] = 0.042
*wR*(*F* ^2^) = 0.116
*S* = 1.08
834 reflections
100 parameters
H-atom parameters constrained
Δρ_max_ = 0.25 e Å^−3^
Δρ_min_ = −0.23 e Å^−3^

### 

Data collection: *SMART* (Siemens, 1996 ▶); cell refinement: *SMART* and *SAINT* (Siemens, 1994 ▶); data reduction: *XPREP* (Siemens, 1994 ▶); program(s) used to solve structure: *SHELXTL* (Sheldrick, 2008 ▶); program(s) used to refine structure: *SHELXTL*; molecular graphics: *SHELXTL*; software used to prepare material for publication: *SHELXTL*.

## Supplementary Material

Crystal structure: contains datablocks I, global. DOI: 10.1107/S1600536810049676/bx2320sup1.cif

Structure factors: contains datablocks I. DOI: 10.1107/S1600536810049676/bx2320Isup2.hkl

Additional supplementary materials:  crystallographic information; 3D view; checkCIF report

## Figures and Tables

**Table 1:** Hydrogen-bond geometry (Å, °)

*D*—H⋯*A*	*D*—H	H⋯*A*	*D*⋯*A*	*D*—H⋯*A*
N1—H1*A*⋯O1^i^	0.86	2.17	2.918 (3)	146
N3—H3*A*⋯O2^i^	0.86	2.06	2.870 (4)	157
N3—H3*B*⋯O2^ii^	0.86	1.95	2.788 (4)	163
N2—H2*A*⋯O1	0.86	2.19	2.601 (3)	109

Symmetry codes: (i) 
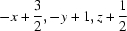
; (ii) 
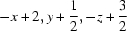
.

**Fig. 1.**
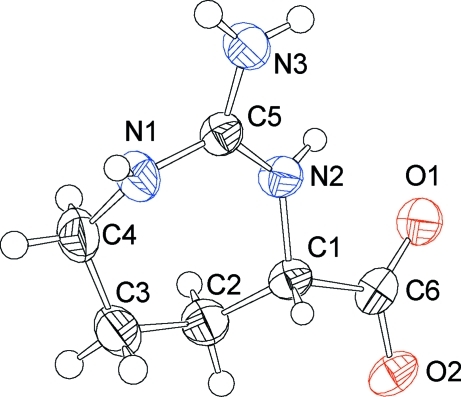
A view of (I) with 50% probability displacement ellipsoids; H atoms are shown as small spheres of arbitrary radii.

**Fig. 2.**
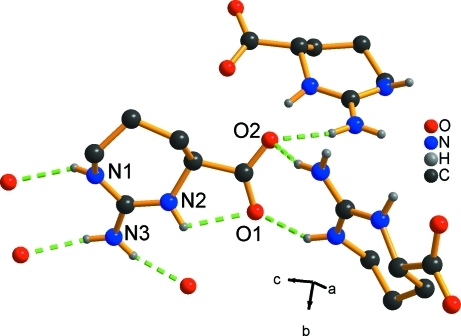
The hydrogen bonding interactions between the molecules. The H- atoms not involved in hydrogen bond are omitted for charity

## supplementary crystallographic information

### Comment

The title compound (I) was hydrothermally synthesized from *L*-Arginine *via* an unusual annulation reaction. We report here a new annulation product derived from the linear arginine molecule. The title compound is the cyclic form of *L*-Arginine and this molecule is a zwitterion with the positive and negative charge residing in the guanidinium and carboxylate groups respectively.The conformation of 1,3-diazepane ring is close to twisted chair.One intramolecular and three intermolecular N—H···O hydrogen bonds stabilize the molecular conformation and the crystal structure respectively (Fig. 2). The C—N distances in the guanidinium group are obviously shorter than that of the normal C—N single bond, indicating delocalized bond of the guanidinium group.

### Experimental

A mixture of Cu(ClO_4_)_2_^.^6H_2_O (0.186 g, 0.5 mmol), *L*-Arginine (0.087 g, 0.5 mmol) and water (10 ml) was sealed in a 15 ml teflon-lined stainless steel reactor and heated to 423 K for 60 h. Colorless crystals of (I) suitable for X-ray analysis were obtained.

### Refinement

All H atoms were placed at calculated positions, and refined with isotropic displacement parameters, using a riding model [C—H = 0.97 Å and *U*_iso_(H) = 1.2*U*_eq_(C), C—H = 0.98 Å and *U*_iso_(H) = 1.5*U*_eq_(C), N—H = 0.86 Å and *U*_iso_(H) = 1.2*U*_eq_(N)].

### Crystal data

**Table:**

C_6_H_11_N_3_O_2_	*F*(000) = 336
*M**_r_* = 157.18	*D*_x_ = 1.353 Mg m^−^^3^
Orthorhombic, *P*2_1_2_1_2_1_	Mo *K*α radiation, λ = 0.71073 Å
Hall symbol: P 2ac 2ab	Cell parameters from 1112 reflections
*a* = 6.1740 (3) Å	θ = 2.7–20.9°
*b* = 8.7979 (5) Å	µ = 0.10 mm^−^^1^
*c* = 14.2036 (7) Å	*T* = 293 K
*V* = 771.51 (7) Å^3^	Prism, colorless
*Z* = 4	0.23 × 0.15 × 0.10 mm

### Data collection

**Table:**

Siemens SMART CCD area-detector diffractometer	834 independent reflections
Radiation source: fine-focus sealed tube	694 reflections with *I* > 2σ(*I*)
graphite	*R*_int_ = 0.026
phi and ω scans	θ_max_ = 25.1°, θ_min_ = 2.7°
Absorption correction: multi-scan (*SADABS*; Sheldrick, 1996)	*h* = −7→5
*T*_min_ = 0.773, *T*_max_ = 1.000	*k* = −9→10
3426 measured reflections	*l* = −16→13

### Refinement

**Table:**

Refinement on *F*^2^	Primary atom site location: structure-invariant direct methods
Least-squares matrix: full	Secondary atom site location: difference Fourier map
*R*[*F*^2^ > 2σ(*F*^2^)] = 0.042	Hydrogen site location: inferred from neighbouring sites
*wR*(*F*^2^) = 0.116	H-atom parameters constrained
*S* = 1.08	*w* = 1/[σ^2^(*F*_o_^2^) + (0.0573*P*)^2^ + 0.2708*P*] where *P* = (*F*_o_^2^ + 2*F*_c_^2^)/3
834 reflections	(Δ/σ)_max_ = 0.002
100 parameters	Δρ_max_ = 0.25 e Å^−^^3^
0 restraints	Δρ_min_ = −0.23 e Å^−^^3^

### Special details

**Table:**

Geometry. All e.s.d.'s (except the e.s.d. in the dihedral angle between two l.s. planes) are estimated using the full covariance matrix. The cell e.s.d.'s are taken into account individually in the estimation of e.s.d.'s in distances, angles and torsion angles; correlations between e.s.d.'s in cell parameters are only used when they are defined by crystal symmetry. An approximate (isotropic) treatment of cell e.s.d.'s is used for estimating e.s.d.'s involving l.s. planes.
Refinement. Refinement of *F*^2^ against ALL reflections. The weighted *R*-factor *wR* and goodness of fit *S* are based on *F*^2^, conventional *R*-factors *R* are based on *F*, with *F* set to zero for negative *F*^2^. The threshold expression of *F*^2^ > σ(*F*^2^) is used only for calculating *R*-factors(gt) *etc*. and is not relevant to the choice of reflections for refinement. *R*-factors based on *F*^2^ are statistically about twice as large as those based on *F*, and *R*- factors based on ALL data will be even larger.

### Fractional atomic coordinates and isotropic or equivalent isotropic displacement parameters (Å^2^)

**Table:**

	*x*	*y*	*z*	*U*_iso_*/*U*_eq_	
O1	0.9643 (4)	0.4869 (3)	0.67888 (15)	0.0471 (7)	
O2	0.8529 (4)	0.2509 (2)	0.64723 (15)	0.0463 (7)	
N1	0.5689 (4)	0.4568 (3)	0.97693 (19)	0.0413 (8)	
H1A	0.5874	0.4365	1.0356	0.050*	
N2	0.7752 (5)	0.5024 (3)	0.84226 (17)	0.0383 (7)	
H2A	0.8317	0.5757	0.8105	0.046*	
N3	0.8387 (5)	0.6355 (3)	0.9771 (2)	0.0492 (8)	
H3A	0.8106	0.6559	1.0350	0.059*	
H3B	0.9404	0.6835	0.9483	0.059*	
C1	0.7420 (6)	0.3583 (4)	0.7926 (2)	0.0352 (8)	
H1B	0.8059	0.2765	0.8303	0.042*	
C2	0.5046 (7)	0.3203 (4)	0.7748 (2)	0.0486 (10)	
H2B	0.4346	0.4071	0.7457	0.058*	
H2C	0.4962	0.2359	0.7310	0.058*	
C3	0.3829 (7)	0.2787 (4)	0.8645 (3)	0.0558 (11)	
H3C	0.2381	0.2455	0.8479	0.067*	
H3D	0.4560	0.1940	0.8946	0.067*	
C4	0.3667 (6)	0.4072 (4)	0.9331 (3)	0.0510 (10)	
H4A	0.3033	0.4936	0.9008	0.061*	
H4B	0.2671	0.3775	0.9826	0.061*	
C5	0.7260 (5)	0.5309 (4)	0.9325 (2)	0.0348 (8)	
C6	0.8659 (6)	0.3684 (4)	0.6983 (2)	0.0372 (8)	

### Atomic displacement parameters (Å^2^)

**Table:**

	*U*^11^	*U*^22^	*U*^33^	*U*^12^	*U*^13^	*U*^23^
O1	0.0583 (16)	0.0522 (14)	0.0306 (12)	−0.0038 (14)	0.0092 (11)	0.0005 (11)
O2	0.0625 (17)	0.0467 (13)	0.0299 (11)	0.0092 (13)	0.0014 (13)	−0.0071 (10)
N1	0.0450 (18)	0.0527 (17)	0.0261 (13)	−0.0089 (15)	0.0052 (13)	0.0025 (13)
N2	0.0500 (18)	0.0391 (14)	0.0257 (13)	−0.0051 (14)	0.0053 (12)	−0.0024 (12)
N3	0.057 (2)	0.0567 (18)	0.0343 (14)	−0.0191 (17)	0.0114 (15)	−0.0135 (14)
C1	0.043 (2)	0.0356 (15)	0.0264 (16)	0.0019 (16)	−0.0015 (15)	−0.0009 (14)
C2	0.051 (2)	0.057 (2)	0.038 (2)	−0.008 (2)	0.0000 (19)	−0.0098 (17)
C3	0.049 (2)	0.061 (2)	0.057 (2)	−0.017 (2)	0.005 (2)	−0.005 (2)
C4	0.042 (2)	0.063 (2)	0.047 (2)	−0.0080 (19)	0.009 (2)	−0.0011 (18)
C5	0.0395 (19)	0.0370 (16)	0.0279 (15)	0.0017 (17)	0.0017 (15)	0.0010 (14)
C6	0.040 (2)	0.046 (2)	0.0256 (16)	0.0110 (18)	−0.0035 (16)	0.0018 (15)

### Geometric parameters (Å, °)

**Table:**

O1—C6	1.238 (4)	C1—C2	1.524 (6)
O2—C6	1.265 (4)	C1—C6	1.545 (4)
N1—C5	1.329 (4)	C1—H1B	0.9800
N1—C4	1.462 (4)	C2—C3	1.523 (5)
N1—H1A	0.8600	C2—H2B	0.9700
N2—C5	1.340 (4)	C2—H2C	0.9700
N2—C1	1.465 (4)	C3—C4	1.496 (5)
N2—H2A	0.8600	C3—H3C	0.9700
N3—C5	1.317 (4)	C3—H3D	0.9700
N3—H3A	0.8600	C4—H4A	0.9700
N3—H3B	0.8600	C4—H4B	0.9700
			
C5—N1—C4	124.6 (3)	H2B—C2—H2C	107.8
C5—N1—H1A	117.7	C4—C3—C2	113.3 (3)
C4—N1—H1A	117.7	C4—C3—H3C	108.9
C5—N2—C1	126.1 (3)	C2—C3—H3C	108.9
C5—N2—H2A	116.9	C4—C3—H3D	108.9
C1—N2—H2A	116.9	C2—C3—H3D	108.9
C5—N3—H3A	120.0	H3C—C3—H3D	107.7
C5—N3—H3B	120.0	N1—C4—C3	116.5 (3)
H3A—N3—H3B	120.0	N1—C4—H4A	108.2
N2—C1—C2	113.9 (3)	C3—C4—H4A	108.2
N2—C1—C6	107.3 (3)	N1—C4—H4B	108.2
C2—C1—C6	110.2 (3)	C3—C4—H4B	108.2
N2—C1—H1B	108.4	H4A—C4—H4B	107.3
C2—C1—H1B	108.4	N3—C5—N1	120.0 (3)
C6—C1—H1B	108.4	N3—C5—N2	118.1 (3)
C3—C2—C1	112.8 (3)	N1—C5—N2	121.9 (3)
C3—C2—H2B	109.0	O1—C6—O2	126.2 (3)
C1—C2—H2B	109.0	O1—C6—C1	119.0 (3)
C3—C2—H2C	109.0	O2—C6—C1	114.8 (3)
C1—C2—H2C	109.0		

### Hydrogen-bond geometry (Å, °)

**Table:**

*D*—H···*A*	*D*—H	H···*A*	*D*···*A*	*D*—H···*A*
N1—H1A···O1^i^	0.86	2.17	2.918 (3)	146
N3—H3A···O2^i^	0.86	2.06	2.870 (4)	157
N3—H3B···O2^ii^	0.86	1.95	2.788 (4)	163
N2—H2A···O1	0.86	2.19	2.601 (3)	109

Symmetry codes: (i) −*x*+3/2, −*y*+1, *z*+1/2; (ii) −*x*+2, *y*+1/2, −*z*+3/2.
